# Revealing novel CD8^+^ T-cell epitopes from the H5N1 avian influenza virus in HBW/B1 haplotype ducks

**DOI:** 10.1186/s13567-024-01415-6

**Published:** 2024-12-18

**Authors:** Wanlin Jiao, Yingyi Chen, Zimin Xie, Li Zhao, Shanyao Du, Mulin Ma, Ming Liao, Manman Dai

**Affiliations:** 1 National and Regional Joint Engineering Laboratory for Medicament of Zoonosis Prevention and Control, Guangdong Provincial Key Laboratory of Zoonosis Prevention and Control, College of Veterinary Medicine, South China Agricultural University, Guangzhou, 510642 China; 2 Present Address: UK-China Centre of Excellence for Research on Avian Diseases, Guangzhou, 510642 China

**Keywords:** H5N1 AIV, MHC B1 haplotype ducks, CD8^+^ T-cell response, MHC B1-restricted T-cell epitopes

## Abstract

The duck CD8^+^ T-cell response effectively defends against H5N1 highly pathogenic avian influenza virus (HPAIV) infection, but the recognized peptide is rarely identified. Here, we found that the ratio of CD8^+^ T cells and the expression of IFN-γ and cytotoxicity-associated genes, including granzyme A/K, perforin and IL2, at 7 days post-infection in peripheral blood mononuclear cells (PBMCs) from B1 haplotype ducks significantly increased in the context of defending against H5N1 AIV infection in vivo. Moreover, similar results were observed in cultured and sorted H5N1 AIV-stimulated duck CD8^+^ T cells in vitro. Next, we selected 109 epitopes as candidate epitopes on the basis of the MHC-I restriction binding peptide prediction website database and further identified twelve CD8^+^ T-cell epitopes that significantly increased IFN-γ gene expression after stimulating B1 haplotype duck memory PBMCs. In particular, NP_338−346_, NP_473−481_, M_2−10_, PB1_540−548_ and PA_80−88_ were highly conserved in H5N1, H5N6, H5N8, H7N9, and H9N2 AIVs. These findings provide directions for the development of universal T-cell epitope vaccines for AIV in ducks.

**Supplementary Information:**

The online version contains supplementary material available at 10.1186/s13567-024-01415-6.

## Introduction, methods and results

The highly pathogenic avian influenza virus (HPAIV) H5N1 is still circulating in multiple countries and continues to pose challenges to global public health [1]. Avian influenza outbreaks have killed or destroyed nearly 450 million poultry since 2005, with H5N1 being the main culprit [2]. Ducks are known to be “Trojan horses” for spreading H5N1 AIV [3], and H5N1 is highly efficient at transmitting H5N1 between ducks and is highly adaptable to ducks [4]. Therefore, reducing the risk of virus infection in ducks is essential for controlling the spread of H5N1 HPAI. Vaccination is an important strategy for preventing AIV infection [5]. Current commercial vaccines are mainly inactivated vaccines that elicit a neutralizing antibody response against AIV. However, the virus can easily mutate via reassortment or antigenic drift to escape antibody neutralization, rendering these vaccines ineffective [6, 7]. Hence, researchers are directing their efforts toward the development of more immunogenic and cross-protective vaccines against HPAIV infection [6].

The specific CD8^+^ T-cell response directed to relatively conserved epitopes has broader strain coverage and provides longer-lasting immune protection [8, 9]. Our previous study demonstrated the importance of the duck CD8^+^ T-cell response in eliminating H5N1 infection [10]. Consequently, identifying epitopes that can induce a robust H5N1-specific duck CD8^+^ T-cell response is important. CD8^+^ T-cell epitopes are presented by major histocompatibility complex class I (MHC-I) molecules to specific T-cell receptors (TCRs) to mediate the antiviral response of CD8^+^ T cells [11]. The MHC-I alleles are highly polymorphic and can encode different peptide binding motifs, thus presenting various peptide epitopes [12], which makes the identification of CD8^+^ T-cell epitopes highly challenging. Fortunately, some MHC haplotype strains have been generated, which greatly mitigates interference from MHC-I diversity and polymorphisms. For common chicken MHC haplotypes, many studies have successfully identified H5N1 AIV-derived CD8^+^ T-cell epitopes [13–15].

Notably, compared with that in chickens, research on virus epitope-specific CD8^+^ T cells in MHC haplotype ducks is still in the initial stage. The MHC class I region of the duck contains five genes (*UAA*,* UBA*,* UCA*,* UDA*, and* UEA*), and the *UAA* adjacent to the *TAP2* gene is a predominantly expressed locus [16]. On the basis of one high egg production local breed of Shaoxing Drake, four stable strains of MHC-I haplotype ducks (HBW/B1-B4) were bred on the basis of four homozygous genotypes determined by several single nucleotide polymorphisms (SNPs) present in the *UAA* locus [17]. A previous study determined the peptide binding motif of the MHC-I molecule in HBW/B4 ducks and successfully identified epitopes of MHC B4-restricted Tembusu virus (TMUV)-specific CD8^+^ T cells [18]. Nonetheless, the role of the CD8^+^ T response against H5N1 AIV in MHC-I haplotype ducks is still unclear, and the AIV-derived epitopes recognized by CD8^+^ T cells are also unknown.

In this study, we investigated the immune response of B1 haplotype ducks to H5N1 in vivo and further studied the CD8^+^ T-cell response by culturing and sorting H5N1-specific CD8^+^ T cells in vitro. Moreover, we used the MHC-I restriction binding peptide prediction website database (NetMHCpan-4.0) to screen the potential MHC class I-restricted T-cell epitopes of H5N1 AIV and further identified the T-cell epitopes by detecting IFN-γ expression in memory PBMCs stimulated with peptides. These findings provide novel directions for the development of vaccines that induce durable immunity.

### H5N1 AIV infection induces duck immune response in vivo

**Figure 1:**
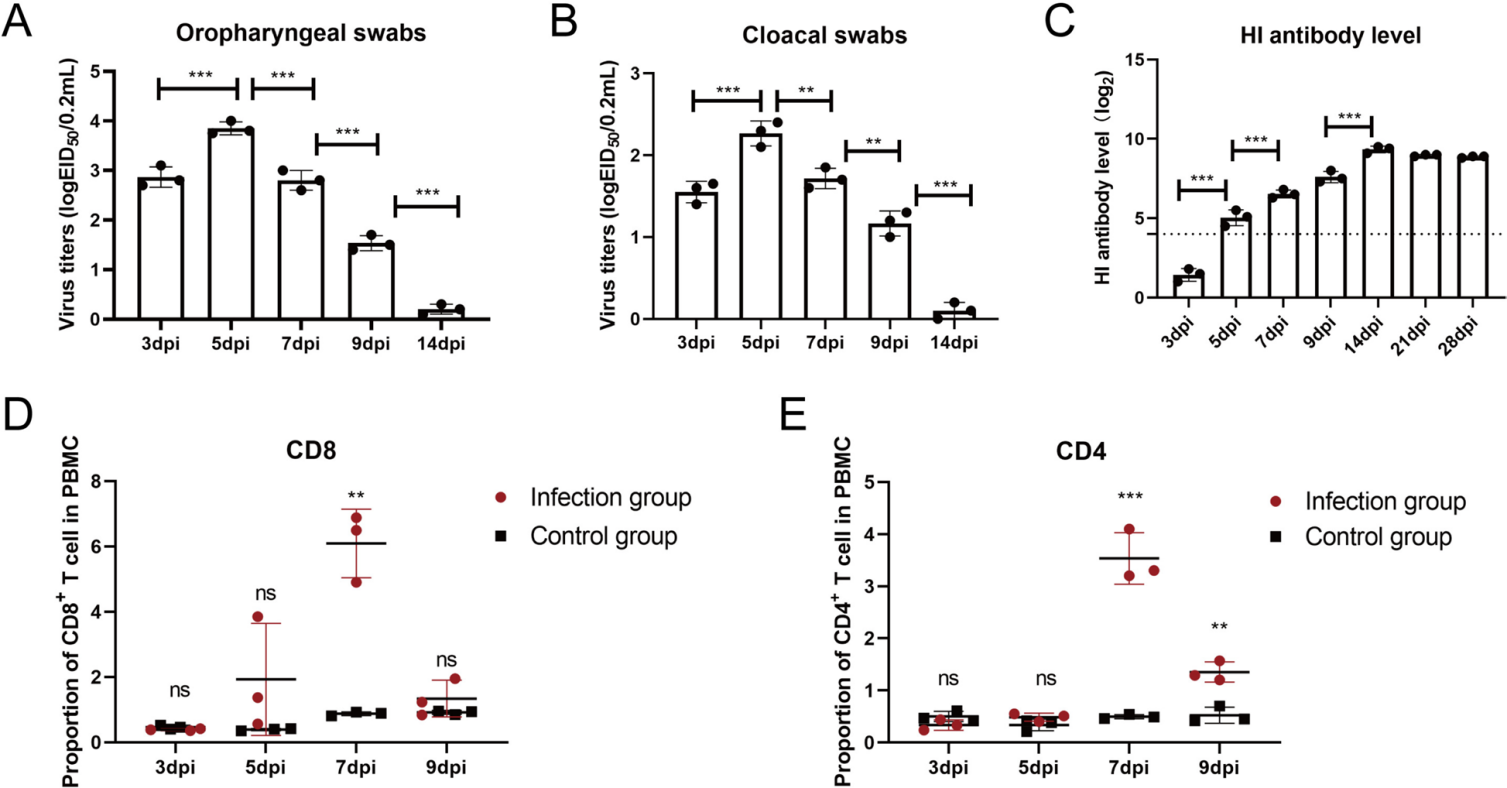
**Monitoring of H5N1 AIV shedding**,** HI Ab levels**,** and T lymphocyte percentages post-infection.** Virus (H5N1 AIV) shedding was monitored via detection of the viral load in oropharyngeal (**A**) and cloacal (**B**) swabs. Statistical analyses of the virus titre in swabs at various time points were performed using one-way ANOVA. **C** HI Ab levels were monitored in 1% chicken RBCs. A value > 4 (dotted line) was considered HI Ab positive. One-way ANOVA was used for statistical comparisons. The percentages of CD8^+^ T cells (**D**) and CD4^+^ T cells (**E**) in the control group and infection group at various time points were detected. The cells (2 × 10^5^) from each sample were collected for flow cytometric analysis. The results are presented as the means ± SEMs, and the unpaired t test was used for statistical comparison. H5N1 virus shedding and H5N1 HI Ab expression in the control group were negative at various time points (data not shown). Three ducks in the infected and control groups were randomly selected for sampling and detection. *P < 0.05, **P < 0.01, ***P < 0.001. ns, not significant.

To study the immune response of B1 haplotype ducks to H5N1 AIV infection in vivo, two-week-old healthy B1 haplotype ducks were purchased from the National Poultry Laboratory Animal Resource Center (affiliated with Harbin Veterinary Research Institute, Chinese Academy of Agricultural Sciences). After being fed for two weeks in negative-pressure isolators, four-week-old B1 haplotype ducks were intranasally inoculated with the H5N1 subtype strain DK383 (A/Duck/Guangdong/383/2008) (10^3.5^ 50% egg infectious dose [EID_50_]/200 µL) [19] and appeared dead within 4 to 6 days post-inoculation (dpi), with an approximately 43% survival rate (data not shown). The oropharyngeal and cloacal swabs of infected ducks were collected from 3 to 14 dpi, and the viral loads in the swabs were measured by EID_50_ as previously reported [20]. The results revealed that virus shedding peaked at 5 dpi and subsequently decreased (Figures 1A and B). Moreover, the antibody levels of the sera collected from 3 to 28 dpi were tested by hemagglutination inhibition (HI) assays, and the antibody level increased significantly at 5 dpi (*P* < 0.01) and peaked at 14 dpi (Figure 1C). Additionally, B1 haplotype duck PBMCs were isolated from 3 to 9 dpi as previously described [21], and T-cell proportions were analysed by flow cytometry. The gating strategy for duck T cells is shown in Additional file 1. Compared with those in the control group, the percentages of CD8^+^ T cells and CD4^+^ T cells among the PBMCs of the infection group significantly increased at 7 dpi (*P* < 0.01) (Figures 1D and E). Hence, HI antibodies, CD8^+^ T cells and CD4^+^ T cells clearly play important roles in the elimination of H5N1 AIV in vivo.

**Figure 2:**
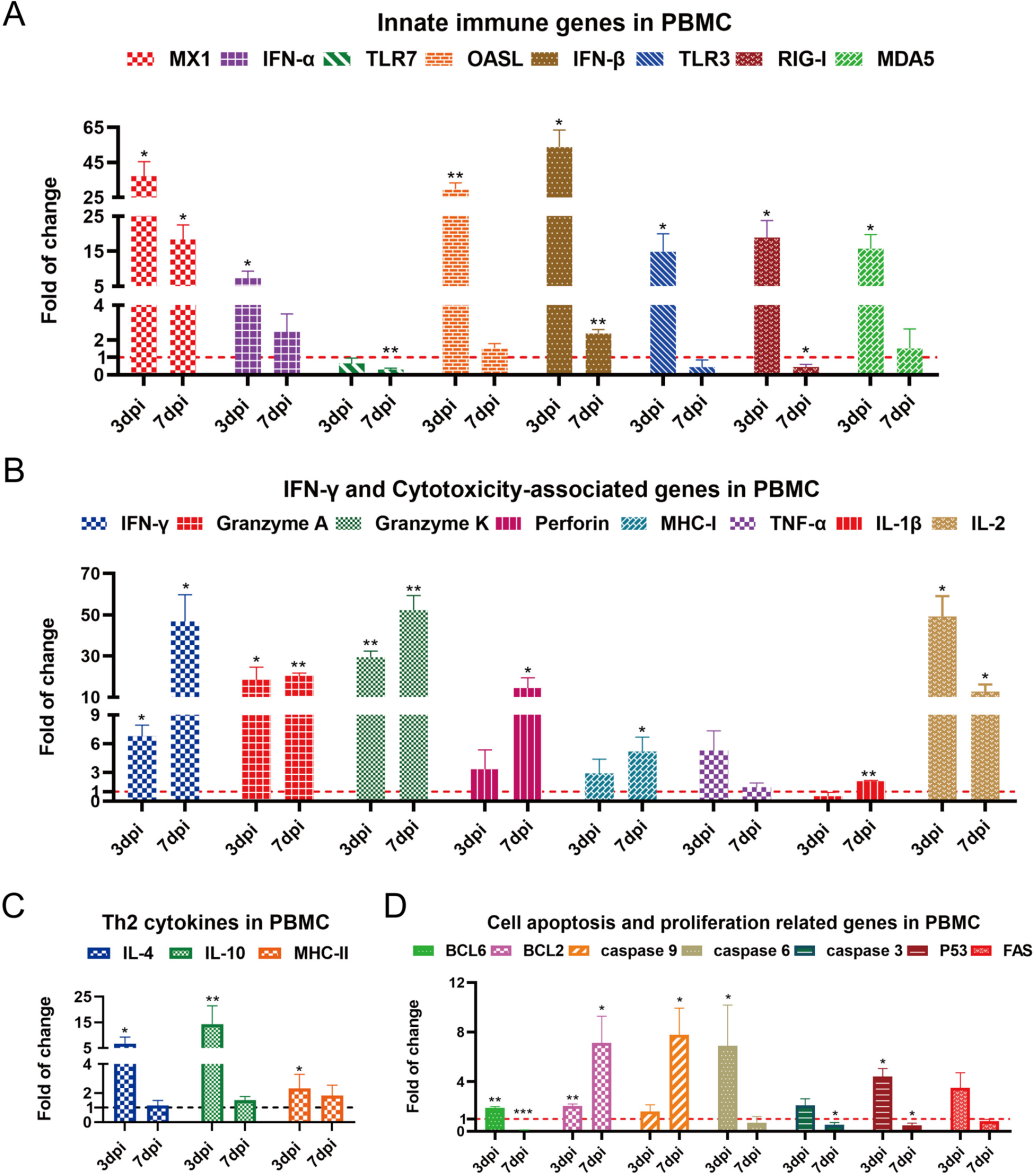
**qRT-PCR analysis of immune-related gene expression in B1 haplotype duck PBMCs after 3 and 7 days of infection**. Total RNA was extracted from the PBMCs of three ducks in the infected and control groups. The data were collected from three biological samples in each group; each sample was analysed in triplicate. **A** Innate immune genes in PBMCs. **B** IFN-γ and cytotoxicity-associated genes in PBMCs. **C** Th2 cytokines in PBMCs. **D** Cell apoptosis- and proliferation-related genes in PBMCs. The results are presented as the means ± SEMs, and paired t tests were used for statistical comparisons. *P < 0.05, **P < 0.01, ***P < 0.001.

Additionally, the expression of immune-related genes in B1 haplotype duck PBMCs was detected via qRT-PCR [22]. Innate immune gene expression, including that of pattern recognition receptors (TLR3, RIG-I and MDA5), the interferon-associated genes IFN-α and IFN-β, and the antiviral-related genes MX1 and OASL at 3 dpi (*P* < 0.05 or 0.01), was significantly increased after H5N1 AIV infection (Figure 2A). Moreover, the mRNA expression levels of IFN-γ and cytotoxicity-associated genes, including granzyme A/K, IL-2 and perforin, at 7 dpi (*P* < 0.05 or 0.01) were significantly elevated in infected ducks (Figure 2B). In addition, the transcript levels of Th2 cytokines (IL-4, IL-10 and MHC-II) at 3 dpi (*P* < 0.05 or 0.01) (Figure 2C) and apoptosis and proliferation genes, including BCL6, caspase 6 and P53 at 3 dpi (*P* < 0.05 or 0.01) and BCL2 and caspase 9 at 7 dpi (*P* < 0.05) (Figure 2D), were significantly increased after H5N1 AIV infection.

### In vitro expansion and detection of H5N1 AIV-specific B1 haplotype duck CD8^+^ T cells

**Figure 3:**
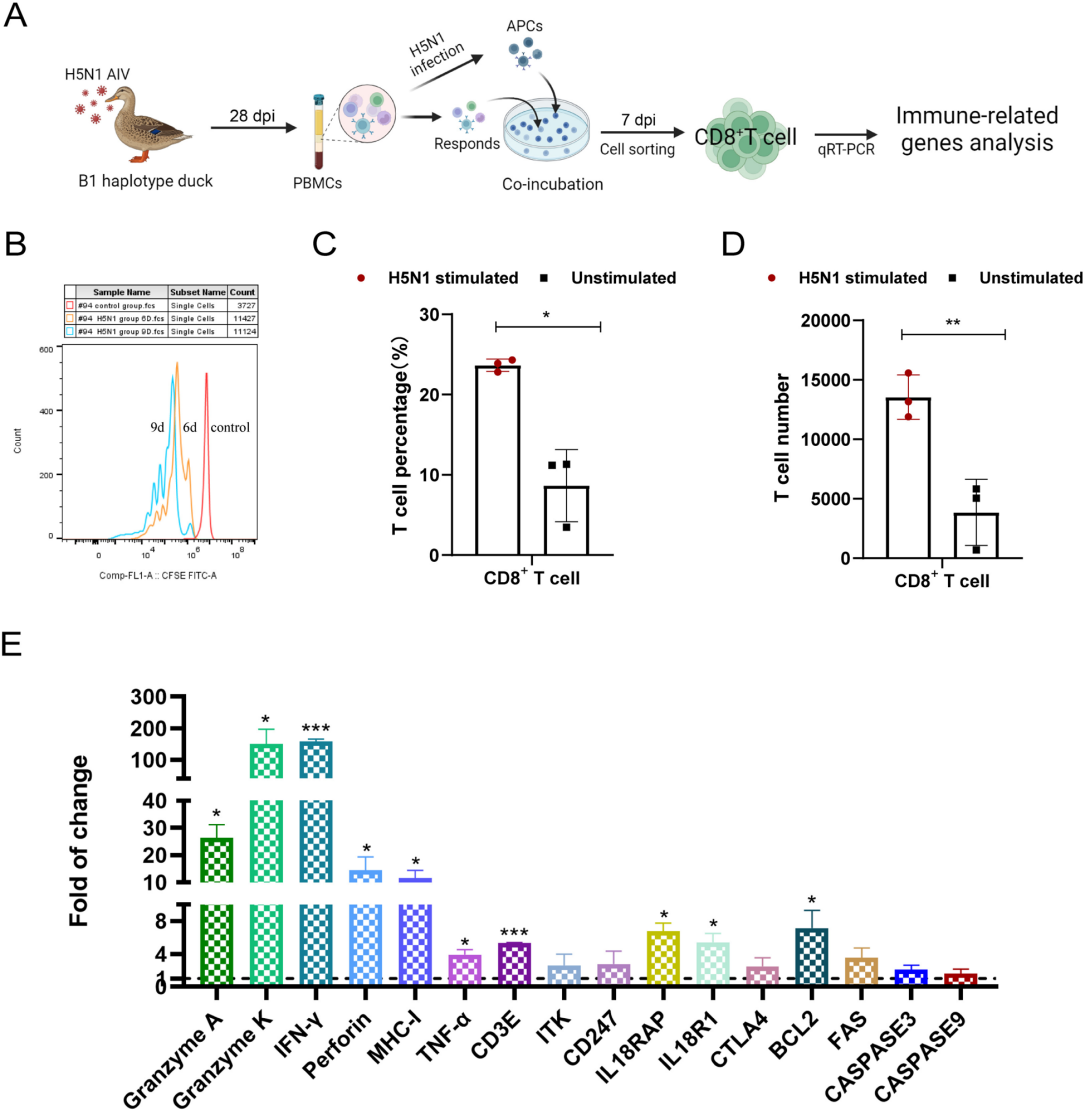
**In vitro culture and response of H5N1 AIV-stimulated CD8**^**+**^**T cells from B1-line ducks.** **A** Diagram of in vitro culture and response detection of H5N1 avian influenza virus-specific duck CD8^+^ T cells. **B** Flow cytometry detection of the proliferation of CFSE-labelled PBMCs stimulated with H5N1 AIV. The red sample indicates CFSE-labelled memory PBMCs without stimulation. The orange and blue samples represent CFSE-labelled PBMCs cultured for 6 and 9 days after H5N1 AIV stimulation, respectively. The percentage of CD8^+^ T cells (**C**) and the number of CD8^+^ T cells (**D**) in H5N1-stimulated and unstimulated cells after 7 days of culture. CD8^+^ T-cell percentage or number data were collected from three replicates in two independent experiments. The results are presented as the means ± SEM, and the unpaired t test was used for statistical comparison. **E** Changes in the transcription of H5N1 AIV-specific CD8^+^ T cells were detected via qRT-PCR. CD8^+^ T cells were sorted from H5N1-stimulated and unstimulated PBMCs after 7 days of culture. The data were collected from three biological samples in triplicate from each H5N1-stimulated and unstimulated group. The results are presented as the means ± SEM, and paired t tests were used for statistical comparisons. *P < 0.05, **P < 0.01, ***P < 0.001.

To investigate the B1 haplotype duck CD8^+^ T-cell response to H5N1 AIV infection, H5N1 AIV-specific B1 haplotype duck T cells were cultured in vitro as previously described [10]. As shown in Figure 3A, we used H5N1 (MOI of 5)-infected or PBS-treated autologous PBMCs as antigen-presenting cells (APCs) to stimulate PBMCs (responders) isolated from infected ducks at 28 dpi. Compared with unstimulated cells (treated with PBS), H5N1 AIV-infected APC-stimulated cells proliferated vigorously and produced distinct cell clusters (Additional file 2). We also used CFSE dilution as an indicator of cell division [23] and further verified H5N1-stimulated cell proliferation at 6 days and 9 days in culture (Figure 3B). Moreover, flow cytometry revealed that the proportion and number of CD8^+^ T cells in H5N1-stimulated cells were significantly greater than those in unstimulated cells after 7 days in culture (Figures 3C and D). Collectively, these results indicate successful expansion of H5N1-specific duck CD8^+^ T cells in vitro.

To further verify the anti-H5N1 AIV effect on PBMC-derived CD8^+^ T cells, we quantitatively analysed the mRNA levels of immune-related genes in CD8^+^ T cells sorted from the above cultured H5N1-stimulated cells or unstimulated cells after 7 days (Figure 3A). The transcript levels of cytotoxicity-associated genes (granzyme A, granzyme K, perforin) (*P* < 0.05), IFN-γ, and CD3E (*P* < 0.001), as well as IL-18RAP, IL-18R1, BCL2, and MHC-I (*P* < 0.05), were significantly increased after H5N1 AIV-stimulated duck CD8^+^ T-cell activation in vitro (Figure 3E). As a consequence, H5N1-specific CD8^+^ T cells produce a significant cytotoxic T-cell response after H5N1 AIV stimulation in vitro.

### Screening of B1 haplotype-restricted H5N1-specific CD8^+^ T-cell epitopes

**Figure 4:**
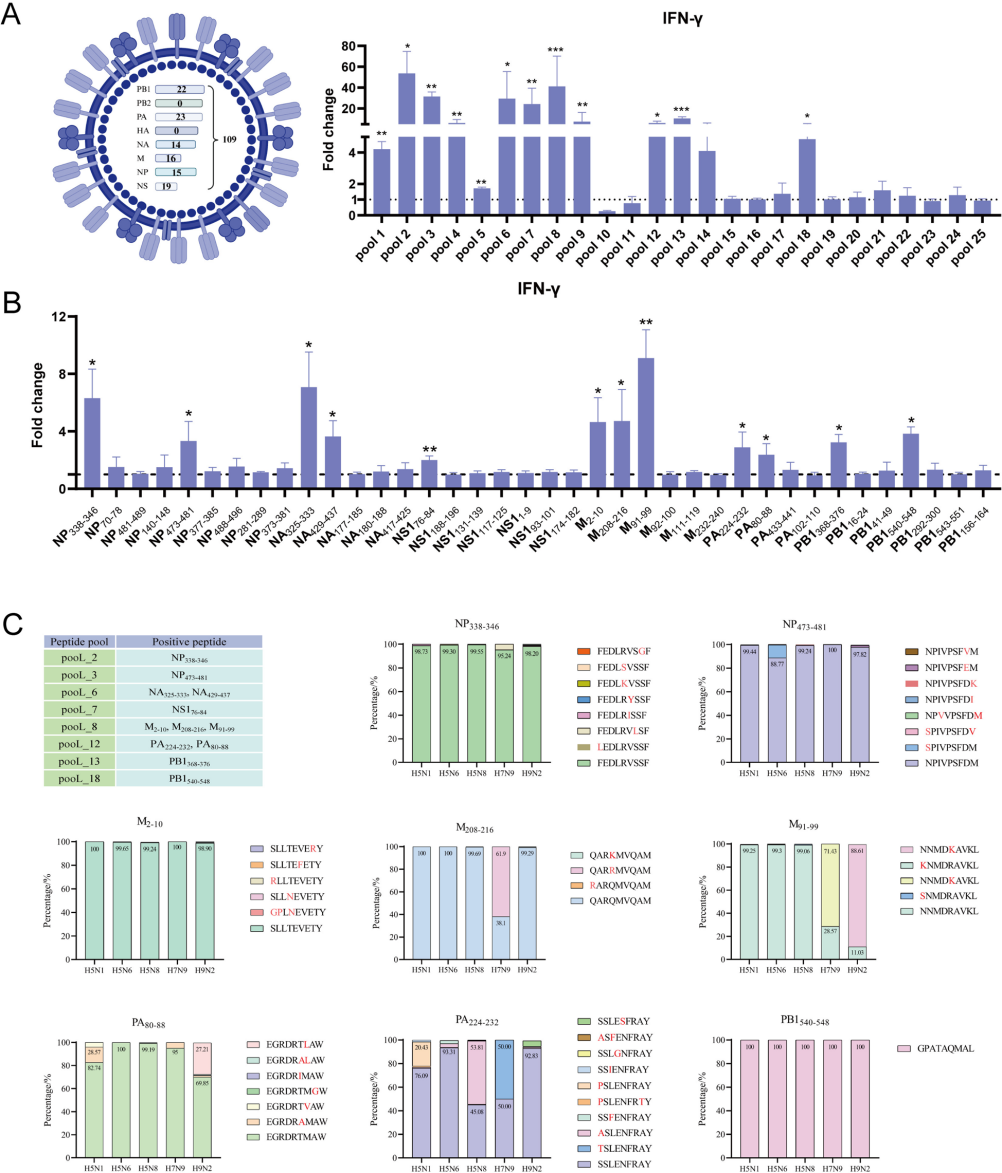
**Screening of immunodominant epitopes of H5N1 AIV-specific CD8**^**+ **^**T cells.** **A** RT-PCR analysis of IFN-γ gene expression in B1 haplotype memory PBMCs stimulated with peptide pools. The data were collected from three replicates of three biological samples. **B** RT-PCR analysis of IFN-γ gene expression in B1 haplotype memory PBMCs with individual 9-mer peptide stimulation from positive peptide pools. The results are presented as the means ± SEM, and paired t tests were used for statistical comparisons. *P < 0.05, **P < 0.01, ***P < 0.001. **C** Conservation of the sequences between circulating strains for the positive peptides. The Global Initiative of Sharing All Influenza Data (GISAID) (gisaid.org) was used with the search criteria set as Asia, 2019 to 2024, NA/NP/M/PB1/PA/NS1, and H5N1/H5N6/H5N8/H7N9/H9N2. Protein sequences were aligned with the MUSCLE algorithm. The frequency of mutation was determined. The protein sequences of influenza A (H5N1-H5N6-H5N8-H7N9-H9N2) viruses are represented by various colored bars, and the number above the bars indicates the number of virus strains.

Eight genome sequences of H5N1 AIV were amplified using the Hoffmann universal primers, and their protein sequences were confirmed by sequencing. The MHC-I restriction binding peptide prediction website database (NetMHCpan-4.0-Services-DTUHealthTech) was subsequently used to predict H5N1-derived MHC-Ι-restricted T-cell epitopes [24]. The predictions revealed 109 candidate peptides. Individual peptides of 9 amino acids were synthesized (GenScript, Nanjing, China) and dissolved in DMSO. The information of the predicted peptides is shown in Additional file 3. We subsequently pooled 3–5 peptides from the same protein as a peptide pool to stimulate memory PBMCs in B1 haplotype ducks in vitro and detected IFN-γ expression by qRT-PCR. As shown in Figure 4A, pooL_1 ~ pooL_9, pooL_12, pooL_13 and pooL_18 (Additional file 4) can significantly stimulate the memory PBMCs of B1 haplotype ducks to express IFN-γ, indicating that there are immunogenic epitopes in the peptide segment comprising the above peptide pools. Therefore, we used the same method to fine-screen individual peptides from pooL_1 ~ pooL_9, pooL_12 ~ pooL_13 and pooL_18. As shown in Figure 4B, compared with the control group, M_91−99_, NS1_76−84_ (*P* < 0.01) and NP_338−346_, NP_473−481_, NA_325−333_, NA_429−437_, M_2−10_, M_208−216_, PA_224−232_, PA_80−88_, PB1_368−376_, and PB1_540−548_ (*P* < 0.05) (Additional file 5) significantly increased IFN-γ gene expression after stimulating B1 haplotype duck memory PBMCs. Thus, these twelve 9-mer peptides can be considered B1-restricted T-cell epitopes.

To analyse the conservation of these T-cell epitope candidates, alignment was performed for H5N1, H5N6, H5N8, H7N9, and H9N2 AIV strains reported in Asia from 2019 to 2024. As shown in Figure 4C, NP_338−346_, NP_473−481_, M_2−10,_ PB1_540−548_ and PA_80−88_ were highly conserved in all five avian influenza subtypes. M_208−216_ in the H5N1, H5N6, H5N8 and H9N2 subtypes; M_91−99_ in the H5N1, H5N6, and H5N8 subtypes; and PA_224−−232_ in the H5N1, H5N6, and H9N2 subtypes also presented high levels of conservation. However, NA_325−333_, NA_429−437_, PB1_368−376_ and NS1_76−84_ were not conserved among the five avian influenza subtypes (Additional file 6).

## Discussion

Our previous research revealed that duck cytotoxic T-cell responses play a critical role in eliminating H5N1 infection [10]. Cytotoxic T-cell responses are mediated by presenting epitopes to TCRs via MHC-I molecules [11]. It has been reported that MHC molecules are closely related to resistance and susceptibility to infectious diseases, and different MHC haplotypes exhibit differential resistance to challenge with viruses [25–27]. In this study, we provide evidence for the importance of the MHC class I-restricted T-cell response against H5N1 in B1 haplotype ducks. Specifically, during H5N1 infection in B1 haplotype ducks, the proportion of CD8^+^ T cells and the transcript levels of IFN-γ and cytotoxicity-related genes increased significantly, and similar results were observed in cultured and sorted H5N1 AIV-stimulated duck CD8^+^ T cells in vitro. Additionally, the receptor helper proteins IL18RAP and IL18R1 can induce the production of IFN-γ [28] in CD8^+^ T cells and are significantly upregulated in cultured and sorted CD8^+^ T cells in vitro. These results show that B1 haplotype duck CD8^+^ T cells produced an obvious immune response to H5N1 AIV infection both in vivo and in vitro. In the future, we hope to investigate and compare the differences in the T-cell response mediated by 4 duck MHC haplotypes (B1–B4), which will help us understand the relationship between avian minimal MHC and disease resistance and provide new insights into breeding ducks that are resistant to certain viral diseases.

The identification of MHC-I-restricted epitopes is the basis for further study of the CD8^+^ T-cell antiviral immune response and the development of epitope vaccines. The analysis of MHC-I crystal structures can greatly facilitate the identification of CD8^+^ T-cell epitopes. Thus far, except for the duck MHC-I Anpl-UAA*01 and B4 haplotype duck MHC-I Anpl-UAA*76 [18, 29], the MHC-I crystal structure of other strains has not been determined, which poses a great challenge for the identification of duck CD8^+^ T-cell epitopes. Bioinformatics tools for predicting epitopes, including NetMHCpan, have evolved, and the accuracy of these methods has improved substantially in recent years [24]. Many studies have successfully predicted candidate epitopes with high binding affinity to MHC-I via NetMHCpan [29–31]. In this study, we predicted candidate epitopes via NetMHCpan-4.0 on the basis of protein sequences of H5N1 and B1 haplotype duck MHC-I molecules. To verify the immunogenicity of the predicted peptides, this study extends earlier research on the identification of T-cell epitopes in chickens to ducks [13, 14, 32]. Ultimately, we revealed twelve MHC B1-restricted T-cell epitopes that significantly increased IFN-γ gene expression after stimulating B1 haplotype duck memory PBMCs. Specifically, NP_338−346_, NP_473−481_, M_2−10_, PB1_540−548_ and PA_80−88_ were extremely conserved in all five avian influenza subtypes. Intriguingly, on the basis of epitope information in the Immune Epitope Database (IEDB), we found that NP_338−346_, NP_473−481_ and PB1_540−548_ were also specific to human CD8^+^ T cells [33–35], which indicates that NP_338−346_, NP_473−481_ and PB1_540−548_ are conserved enough to serve as potential universal epitopes for protecting animals and humans.

In conclusion, our study revealed that B1 haplotype duck CD8^+^ T cells respond strongly to H5N1 AIV infection and significantly upregulate IFN-γ and cytotoxicity-associated gene expression in vivo and in vitro. Moreover, we revealed twelve MHC B1-restricted T-cell epitopes that can significantly increase IFN-γ gene expression after stimulating B1 haplotype duck memory PBMCs. Notably, NP_338−346_, NP_473−481_, M_2−10,_ PB1_540−548_ and PA_80−88_ were extremely conserved in all five avian influenza subtypes and can be used as potential universal T-cell epitopes.

## Supplementary Information

**Additional file 1. Gating strategy.** Gating strategy for duck CD4^+^ T cells (A) and CD8^+^ T cells (B) in PBMCs.

**Additional file 2. Morphological observation of memory PBMCs with or without H5N1 AIV stimulation.**

**Additional file 3. Potential immunogenic peptide screening from the database.**

**Additional file 4. Peptides from positive peptide pools.**

**Additional file 5. Information on twelve B1-restricted CD8**^**+**^**T-cell epitopes.**

**Additional file 6. Conservation of the sequences between the circulating strains for NA**_**325−−333**_, **NA**_**429−−437**_, **NS1**_**76−−84**_**and PB1**_**368−−376**_. The Global Initiative of Sharing All Influenza Data (GISAID) (gisaid.org) was used with the search criteria set as Asia, 2019 to 2024, NA/NS1/PB1, and H5N1/H5N6/H5N8/H7N9/H9N2. Protein sequences were aligned using the MUSCLE algorithm. The frequency of mutation was determined. The protein sequences of influenza A (H5N1-H5N6-H5N8-H7N9-H9N2) viruses are represented by various colored bars, and the number above the bars indicates the number of virus strains.
