# Supplementary figures and images for: Revealing novel CD8+ T-cell epitopes from the H5N1 avian influenza virus in HBW/B1 haplotype ducks
Source: Vet Res. 2024 Dec 18;55:169. doi: 10.1186/s13567-024-01415-6 (PMC11653964; doi:10.1186/s13567-024-01415-6)

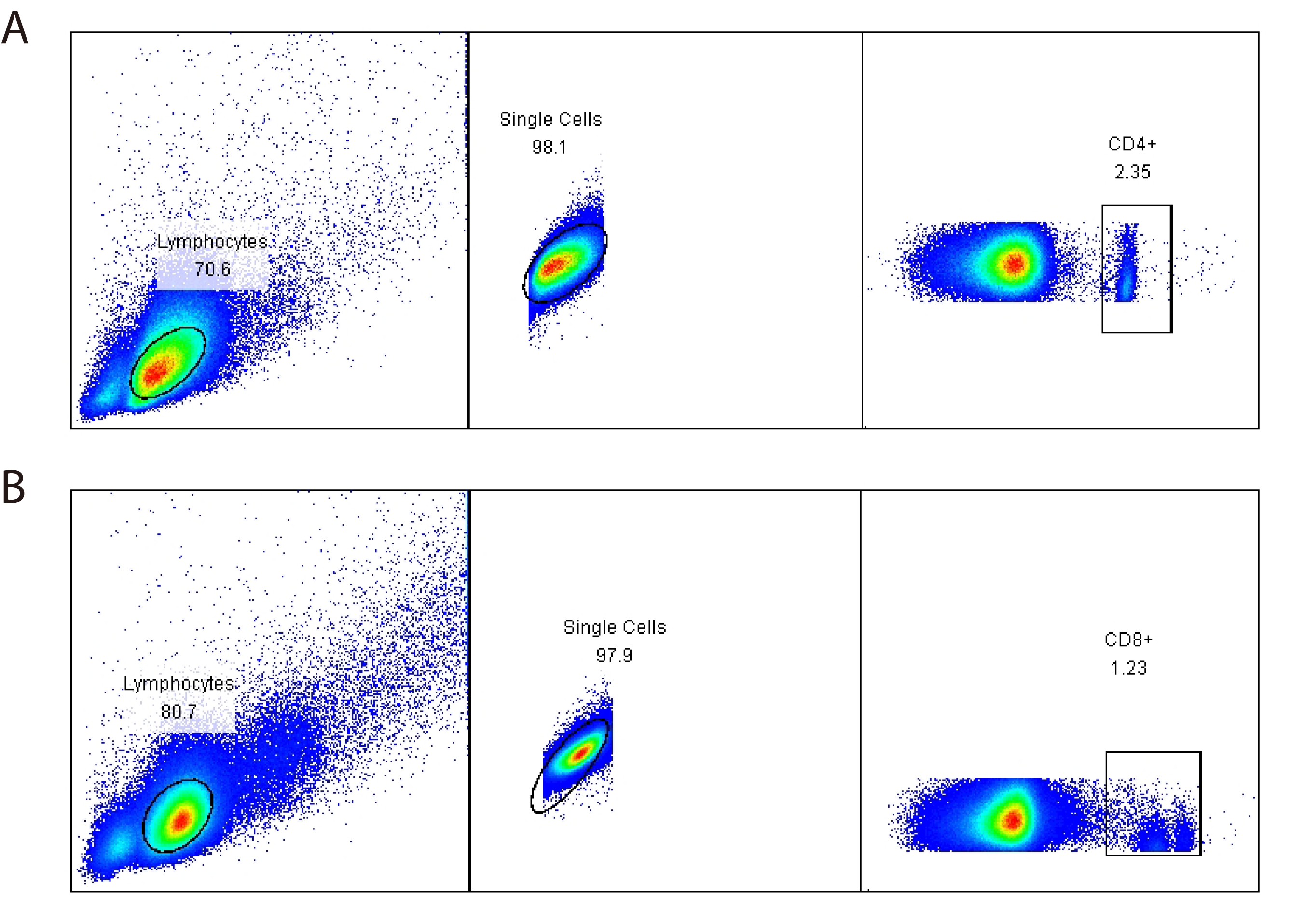

Supplement: Supplementary file 1 — Additional file 1. Gating strategy. Gating strategy for duck CD4+ T cells (A) and CD8+ T cells (B) in PBMCs. [file 13567_2024_1415_MOESM1_ESM.tif]

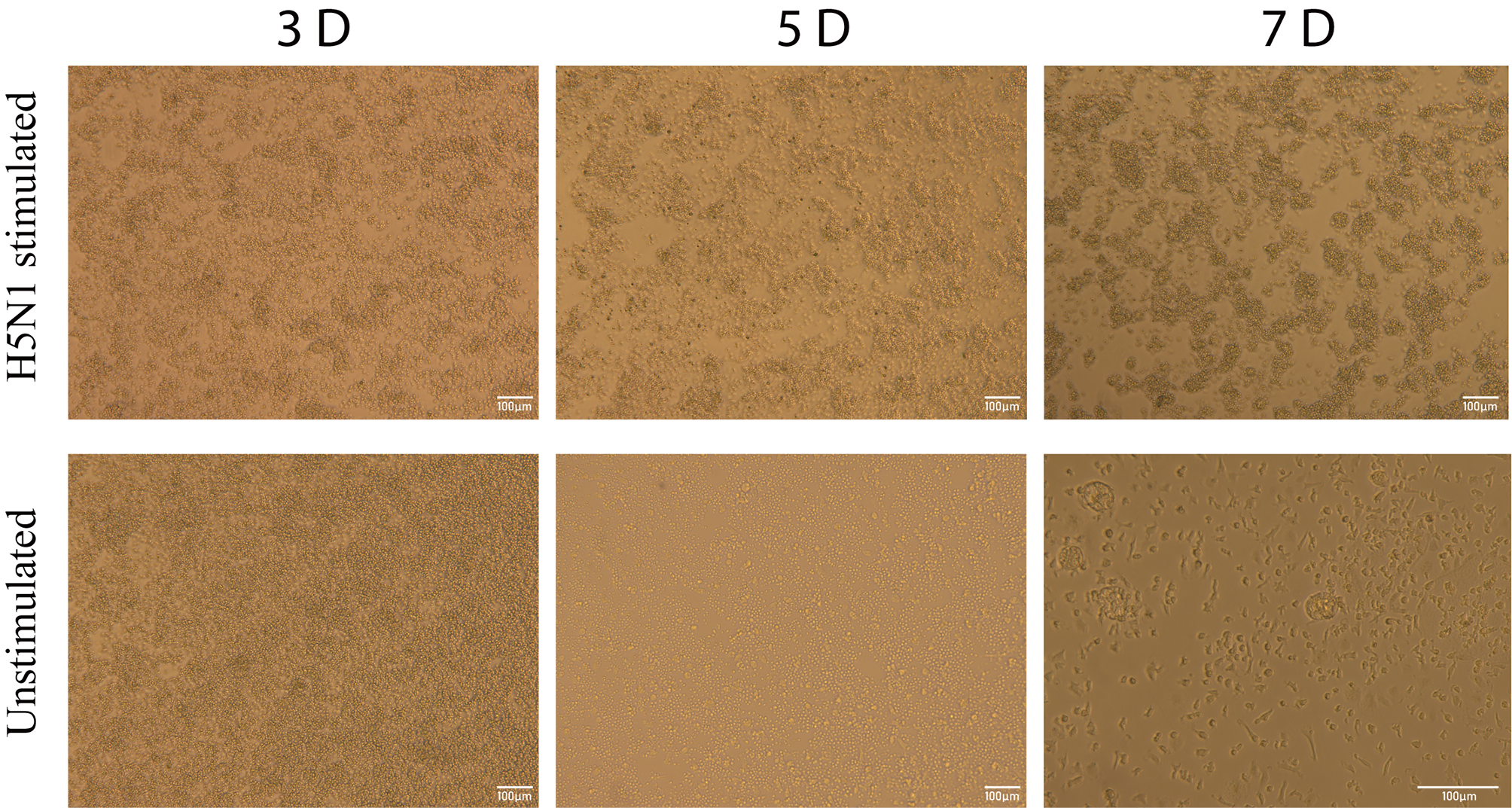

Supplement: Supplementary file 2 — Additional file 2. Morphological observation of memory PBMCs with or without H5N1 AIV stimulation. [file 13567_2024_1415_MOESM2_ESM.tif]

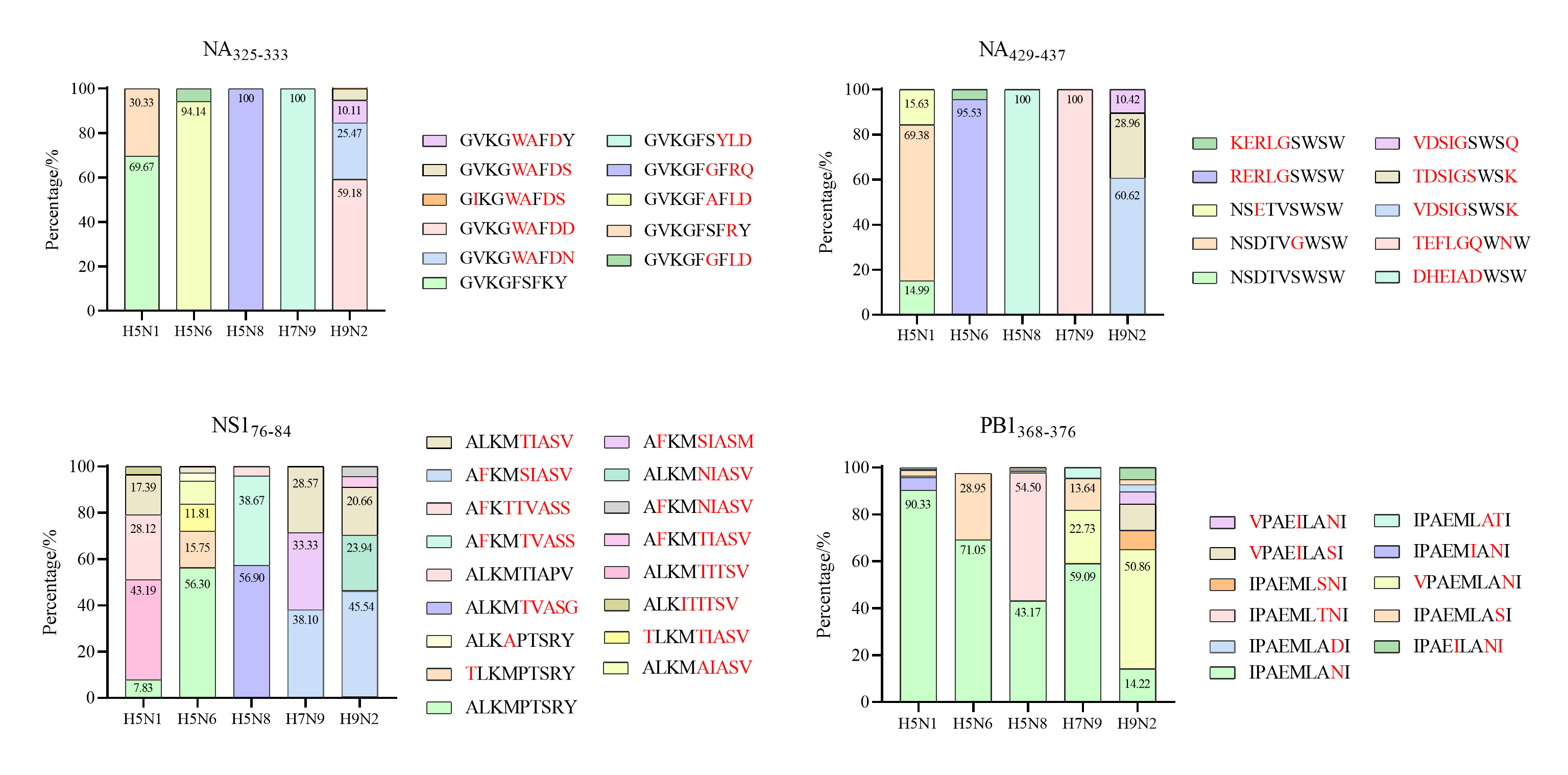

Supplement: Supplementary file 6 — Additional file 6. Conservation of the sequences between the circulating strains for NA325−−333, NA429−−437, NS176−−84and PB1368−−376. The Global Initiative of Sharing All Influenza Data (GISAID) (gisaid.org) was used with the search criteria set as Asia, 2019 to 2024, NA/NS1/PB1, and H5N1/H5N6/H5N8/H7N9/H9N2. Protein sequences were aligned using the MUSCLE algorithm. The frequency of mutation was determined. The protein sequences of influenza A (H5N1-H5N6-H5N8-H7N9-H9N2) viruses are represented by various colored bars, and the number above the bars indicates the number of virus strains. [file 13567_2024_1415_MOESM6_ESM.tif]
