# Supplementary material for: Revealing novel CD8+ T-cell epitopes from the H5N1 avian influenza virus in HBW/B1 haplotype ducks
Source: Vet Res. 2024 Dec 18;55:169. doi: 10.1186/s13567-024-01415-6 (PMC11653964; doi:10.1186/s13567-024-01415-6)
Supplement: Supplementary file 3 — Additional file 3. Potential immunogenic peptide screening from the database. [file 13567_2024_1415_MOESM3_ESM.docx]

**Additional file 3. Potential immunogenic peptides screening from the database.**

| **Viral protein** | **Peptide sequences** | | | |
| --- | --- | --- | --- | --- |
| PB1 | FPYTGDPPY | FIKDYRYTY | APIMFSNKM | VSDGGPNLY |
|  | NASISTTFPY | YSHGTGTGY | VQNAISTTF | HGPAKSMEY |
|  | TPGMQIRGF | DTVNRTHQY | QLFIKDYRY | IPAEMLVNI |
|  | DAVATTHSW | EEMEITTHF | MLVNIDLKY | NSQDTELSF |
|  | GPATAQMAL | TAQMALQLF | TANESGRLM | SPGMMMGMF |
|  | ILNLGQKRY | GTFEFTSFF |  |  |
|  |  |  |  |  |
| PA | APIEHIASM | KPKFLPDLY | SSLENFRAY | NPKIETNKF |
|  | YASPQLEGF | VVNFVSMEF | IASMRRNYF | KTHIHIFSF |
|  | MATKADYTL | GTFDLGGLY | AAMDDFQLI | LAEKAMKEY |
|  | HEGEGIPLY | MEDFVRQCF | RARIKTRLF | VSHCRATEY |
|  | HIASMRRNY | FIDERGESI | EGRDRTMAW | DPRLEPHKW |
|  | VTRREVHIY | HEKGINPNY | SEKTHIHIF |  |
|  |  |  |  |  |
| NA | APSPYNSRF | GPSNGQASY | WPDGAELPF | VSFNQNLEY |
|  | GPVSPNGAY | ELNAPNYHY | NPNQKIITI | VAITDWSGY |
|  | SVELNAPNY | GPDNGAVAV | GVKGFSFKY | NSDTVSWSW |
|  | NGAVAVLKY | WTSGSSISF |  |  |
|  |  |  |  |  |
| NP | LPFERATIM | ATNPIVPSF | MVSGIGRFY | SARPEDVSF |
|  | AFDERRNRY | MNNEGSYFF | HSNLNDATY | FEDLRVSSF |
|  | NTLELRSRY | FFGDNAEEY | YGLAVASGY | NPIVPSFDM |
|  | AMDSNTLEL | DMNNEGSYF | LAVASGYDF |  |
|  |  |  |  |  |
| M | GAKEVALSY | NLLENLQAY | NMDRAVKLY | IAQKLEDIF |
|  | SLLTEVETY | QARQMVQAM | LASCMGLIY | NNMDRAVKL |
|  | NNMDRAVKL | IRHENRMVL | LRDNLLENL | MEWLKTRPI |
|  | QRRRFVQNA | KREITFHGA | RRRFVQNAL | EQAAEAMEV |
|  |  |  |  |  |
| NS1 | RVSETIQRF | VIFDRLETL | AIMDKTIIL | ALKMPTSRY |
|  | MDSNTVSSF | MSRDWFMLM | GVLIGGLEW |  |
|  |  |  |  |  |
| NS2 | MDSNTITSF | SFQDILQRM | SLQNRNATW | DLNGMITQF |
|  | TQFERLKIY | ESEIRTFSF | NSFEQITEL | EVESEIRTF |
|  | MRMGDLHSL | YRDSLGESM | ITFLQALQL | ILTKTENSF |
