# Supplementary material for: Revealing novel CD8+ T-cell epitopes from the H5N1 avian influenza virus in HBW/B1 haplotype ducks
Source: Vet Res. 2024 Dec 18;55:169. doi: 10.1186/s13567-024-01415-6 (PMC11653964; doi:10.1186/s13567-024-01415-6)
Supplement: Supplementary file 4 — Additional file 4. Peptides from positive peptide pools. [file 13567_2024_1415_MOESM4_ESM.docx]

**Additional file 4. Peptides from positive peptide pools.**

| Peptide pool | Peptide name |
| --- | --- |
| pooL_1 | NP_418-426_, NP_471-479_, NP_32-40_, NP_450-458_ |
| pooL_2 | NP_70-78_, NP_481-489_, NP_140-148_, NP_338-346_ |
| pooL_3 | NP_377-385_, NP_488-496_, NP_281-289_, NP_473-481_, NP_373-381_ |
| pooL_4 | NA_146-154_, NA_225-233_, NA_437-445_, NA_284-292_, NA_316-324_ |
| pooL_5 | NA_248-256_, NA_2-10_, NA_374-382_, NA_246-254_ |
| pooL_6 | NA_177-185_, NA_325-333_, NA_429-437_, NA_180-188_, NA_417-425_ |
| pooL_7 | NS1_188-196_, NS1_131-139_, NS1_117-125_, NS1_76-84_, NS1_1-9_, NS1_93-101_, NS1_174-182_ |
| pooL_8 | M_111-119_, M_232-240_, M_92-100_, M_2-10_, M_208-216_, M_91-99_ |
| pooL_9 | NS2_8-16_, NS2_57-65_, NS2_27-35_, NS2_33-41_ |
| pooL_12 | PA_80-88_, PA_433-441_, PA_102-110_, PA_224-232_ |
| pooL_13 | PB1_368-376_, PB1_16-24_, PB1_41-49_ |
| pooL_18 | PB1_292-300_, PB1_540-548_, PB1_543-551_, PB1_156-164_ |
