# Supplementary material for: Revealing novel CD8+ T-cell epitopes from the H5N1 avian influenza virus in HBW/B1 haplotype ducks
Source: Vet Res. 2024 Dec 18;55:169. doi: 10.1186/s13567-024-01415-6 (PMC11653964; doi:10.1186/s13567-024-01415-6)
Supplement: Supplementary file 5 — Additional file 5. Information on twelve B1-restricted CD8+T-cell epitopes. [file 13567_2024_1415_MOESM5_ESM.docx]

**Additional file 5. Information on twelve B1-restricted CD8^+^ T cell epitopes.**

| Epitope name | Sequence | Viral protein | Protein locus |
| --- | --- | --- | --- |
| NA_325-333_ | GVKGFSFKY | NA | 325-333 |
| NA_429-437_ | NSDTVSWSW | NA | 429-437 |
| NP_338-346_ | FEDLRVSSF | NP | 338-346 |
| NP_473-481_ | NPIVPSFDM | NP | 473-481 |
| M_2-10_ | SLLTEVETY | M | 2-10 |
| M_208-216_ | QARQMVQAM | M | 208-216 |
| M_91-99_ | NNMDRAVKL | M | 91-99 |
| NS1_76-84_ | ALKMPTSRY | NS1 | 76-84 |
| PB1_368-376_ | IPAEMLVNI | PB1 | 368-376 |
| PB1_540-548_ | GPATAQMAL | PB1 | 540-548 |
| PA_224-232_ | SSLENFRAY | PA | 224-232 |
| PA_80-88_ | EGRDRTMAW | PA | 80-88 |
